# Identifying Person-Specific Drivers of Depression in Adolescents: Protocol for a Smartphone-Based Ecological Momentary Assessment and Passive Sensing Study

**DOI:** 10.2196/43931

**Published:** 2024-07-16

**Authors:** Mei Yi Ng, Jennifer A Frederick, Aaron J Fisher, Nicholas B Allen, Jeremy W Pettit, Dana L McMakin

**Affiliations:** 1 Department of Psychology and Center for Children and Families Florida International University Miami, FL United States; 2 Department of Psychology University of California, Berkeley Berkeley, CA United States; 3 Department of Psychology University of Oregon Eugene, OR United States

**Keywords:** adolescents, depression, idiographic assessment, network modeling, treatment personalization, ecological momentary assessment, mobile sensing, digital phenotyping, actigraphy, smartphones

## Abstract

**Background:**

Adolescence is marked by an increasing risk of depression and is an optimal window for prevention and early intervention. Personalizing interventions may be one way to maximize therapeutic benefit, especially given the marked heterogeneity in depressive presentations. However, empirical evidence that can guide personalized intervention for youth is lacking. Identifying person-specific symptom drivers during adolescence could improve outcomes by accounting for both developmental and individual differences.

**Objective:**

This study leverages adolescents’ everyday smartphone use to investigate person-specific drivers of depression and validate smartphone-based mobile sensing data against established ambulatory methods. We describe the methods of this study and provide an update on its status. After data collection is completed, we will address three specific aims: (1) identify idiographic drivers of dynamic variability in depressive symptoms, (2) test the validity of mobile sensing against ecological momentary assessment (EMA) and actigraphy for identifying these drivers, and (3) explore adolescent baseline characteristics as predictors of these drivers.

**Methods:**

A total of 50 adolescents with elevated symptoms of depression will participate in 28 days of (1) smartphone-based EMA assessing depressive symptoms, processes, affect, and sleep; (2) mobile sensing of mobility, physical activity, sleep, natural language use in typed interpersonal communication, screen-on time, and call frequency and duration using the Effortless Assessment of Risk States smartphone app; and (3) wrist actigraphy of physical activity and sleep. Adolescents and caregivers will complete developmental and clinical measures at baseline, as well as user feedback interviews at follow-up. Idiographic, within-subject networks of EMA symptoms will be modeled to identify each adolescent’s person-specific drivers of depression. Correlations among EMA, mobile sensor, and actigraph measures of sleep, physical, and social activity will be used to assess the validity of mobile sensing for identifying person-specific drivers. Data-driven analyses of mobile sensor variables predicting core depressive symptoms (self-reported mood and anhedonia) will also be used to assess the validity of mobile sensing for identifying drivers. Finally, between-subject baseline characteristics will be explored as predictors of person-specific drivers.

**Results:**

As of October 2023, 84 families were screened as eligible, of whom 70% (n=59) provided informed consent and 46% (n=39) met all inclusion criteria after completing baseline assessment. Of the 39 included families, 85% (n=33) completed the 28-day smartphone and actigraph data collection period and follow-up study visit.

**Conclusions:**

This study leverages depressed adolescents’ everyday smartphone use to identify person-specific drivers of adolescent depression and to assess the validity of mobile sensing for identifying these drivers. The findings are expected to offer novel insights into the structure and dynamics of depressive symptomatology during a sensitive period of development and to inform future development of a scalable, low-burden smartphone-based tool that can guide personalized treatment decisions for depressed adolescents.

**International Registered Report Identifier (IRRID):**

DERR1-10.2196/43931

## Introduction

### Background

Adolescents experience escalating risk of developing clinical depression, with lifetime prevalence rates doubling from age 13 years (8%) to 18 years (15%) [1]. Depression impairs young people’s academic, social, and physical functioning [2]; often continues into adulthood [2]; and increases the risk of developing other disorders [2] and suicide attempts [3]. Puberty brings a host of changes, including significant developmental changes in the brain, physical growth spurts and sexual maturation, metabolic and circadian changes, along with an emerging identity, increasingly complex social contexts, and increased learning capacity [4]. These changes may contribute to the risk of developing depression but also signal an opportunity for high-impact intervention [5]. Mastering mood management skills during adolescence can alter the developmental course of depression, thereby improving long-term outcomes. Unfortunately, existing therapies fail to sufficiently capitalize on this developmental window. Meta-analyses revealed significantly smaller effects of psychotherapy for depressed adolescents than for adults [6] and for youth depression than for anxiety or conduct problems [7]. The efficacy of evidence-based psychotherapies such as cognitive behavioral therapy (CBT) may be limited by their fixed, linear delivery to all individuals. Personalizing intervention selection, sequencing, and combination may maximize therapeutic benefit [8], especially for a disorder as heterogeneous as depression. However, empirical support on how to do so is lacking.

One promising avenue for informing personalization is to identify symptoms that are *influential* in the onset and maintenance of dynamic variability in depressive symptoms (ie, short-term, temporal relationships among symptoms), that are *modifiable* through intervention, and that *vary across individuals—*referred to here as *person-specific drivers of depression*. Most evidence-based approaches to therapy, such as CBT, attempt to teach individuals coping skills that will allow them to moderate these dynamics in real time. As such, understanding these drivers can inform personalized intervention that focuses on the specific skills most relevant to an individual’s own affective dynamics. Extant theory suggests possible drivers, such as negative affect, rumination, avoidance, social isolation, and anhedonia. Empirical research with 3000+ adults with major depression has documented sadness, loss of energy, interest, and pleasure; concentration problems; sympathetic arousal; and panic as the most *central* (ie, strongly interconnected) among a network of depressive and nondepressive symptoms [9]. Moreover, in never-depressed adults, central depressive symptoms prospectively predicted the onset of depression [10]. Importantly, some central symptoms predicted subsequent changes in other symptoms among depressed or anxious adults, and marked heterogeneity was found in these temporally-ordered predictive relationships [11,12], with no 2 persons sharing the exact number or nature of predictive relationships. Similarly, a large internet-based CBT trial showed that >90% of depressed adults had unique patterns of treatment response, with certain symptoms impacted directly by treatment, which then influenced the reduction of other symptoms [13]. Modeling these person-specific patterns may uncover the mechanisms by which symptoms are maintained and resolved with treatment, providing powerful tools for personalizing treatment. Researchers have led efforts to select and sequence CBT modules to target person-specific drivers identified by idiographic models early in therapy [12,14], which is purportedly more efficacious and efficient due to the drivers’ downstream effect on other symptoms. An open trial [15] of personalized CBT returned effect sizes 1.5-2 times larger, achieved over a shorter duration, compared to a historical benchmark [16]. However, no published studies have examined person-specific drivers of depression among adolescents. To our knowledge, only group-level central internalizing symptoms have been studied among youth [17-19]. An idiographic approach is needed to obtain novel insights about symptom dynamics among depressed adolescents in order to inform interventions that account for both developmental and individual differences and that can shift the trajectory of depression onset, maintenance, and recurrence.

To model symptom dynamics, ecological momentary assessment (EMA) is often used [11,20]. Participants are prompted to provide subjective self-report of experiences and events through brief web-based or text surveys daily or repeatedly throughout the day. Whereas EMA involves *active* input from participants, *passive* assessment uses a mobile or wearable device to unobtrusively record participants’ behavior or physiology. Wrist-worn actigraphs are research-grade devices containing accelerometers and sometimes other sensors (eg, a light sensor) to estimate behavioral indices of sleep and physical activity [21], which relate to key symptoms of depression (ie, hypersomnia or insomnia, fatigue, and psychomotor retardation or agitation). Actigraphy has been validated against gold standard lab-based measures of sleep (ie, polysomnography [22]) and physical activity (ie, indirect calorimetry [23]). Smartphone-based EMA and wrist actigraphy have been successfully used with youth [24-26] and can be feasibly used to examine person-specific drivers with depressed adolescents.

Smartphone-based mobile sensing may offer advantages over established methods—especially for adolescents—but needs validation. Mobile sensing or digital phenotyping [27,28] minimizes response burden while leveraging adolescents’ near universal (95%) smartphone ownership [29] and usage to obtain objective, fine-grained data about their everyday behaviors. Systematic reviews [30,31] have documented promising evidence for using mobile sensor data (sometimes with EMA) to monitor mood by measuring behaviors related to depression, including sleep (through light sensors, accelerometers, and screen time), physical activity (through accelerometers), mobility (through GPS), and social activity (through call and text frequency or duration). Moreover, a national study of depressed adults showed that idiographic models of GPS-based mobility and other sensor data predicted daily mood at the individual level but not at the group level [32], suggesting the utility of such data for elucidating person-specific symptom dynamics. However, there are very few published studies using mobile sensing to collect data or monitor mood among depressed youth, and of these studies, participant samples tend to be small (range 11-37) [33-36], and large amounts of missing data that affect prediction modeling have been reported [33-37]. More validation studies—especially those with larger samples of depressed adolescents—are needed to demonstrate that mobile sensor data collected from depressed adolescents are correlated with, or predictive of, their EMA and actigraph data before mobile sensing can supplement or substitute these established methods.

Baseline predictors of person-specific drivers could distinguish subgroups with different treatment targets. Identifying between-subject characteristics that predict within-person drivers could facilitate efficient assessment and treatment decisions. For example, a study examining a network of positive and negative emotions, stress, and physical activity found that high arousal positive affect (eg, enthusiastic) was the strongest driver (ie, reduced stress and negative affect) among nonanhedonic individuals, whereas low arousal positive affect (eg, relaxed) was the strongest driver among anhedonic individuals [38]. These findings suggest different optimal intervention targets for the 2 subgroups of depressed adults. Exploring baseline markers that predict person-specific drivers and within-person symptom dynamics is an initial step toward the development of strategies to tailor interventions to optimize outcomes.

### Objectives

This study will leverage adolescents’ everyday smartphone use for the purpose of evaluating the contributions of these data to understanding person-specific drivers of depression and to further validate smartphone-based mobile sensing data against established ambulatory methods such as EMA and actigraphy. We plan to address three specific aims in this study after data collection is complete. Aim 1: model idiographic, within-subject networks of EMA symptoms to identify person-specific drivers of depression. We hypothesize that most (≥50%) adolescents will have person-specific drivers: (1a) symptoms strongly associated with, or (1b) prospectively predict change in other symptoms in an idiographic network, and (1c) differ markedly across adolescents. Aim 2: (2a) compute correlations among EMA, mobile sensor, and wrist-worn actigraph measures of sleep, physical, and social activity; and (2b) conduct data-driven analyses of core depressive symptoms (ie, self-reported mood and anhedonia) to assess the validity of mobile sensing for identifying person-specific drivers. Aim 3: explore between-subject baseline characteristics as predictors of these person-specific drivers. For this protocol report, the objectives are to describe the methods of this study and provide an update on the status of this study.

## Methods

### Participants and Recruitment

A total of 50 adolescents who are (1) aged between 12 and 18 years, (2) have one parent or legal guardian willing to participate, (3) report elevated levels of depressive symptoms (a score of ≥16 on the Center for Epidemiologic Studies Depression (CES-D) [39]), and (4) own a smartphone will be included in the study. The targeted age range covers the period when depression rates start to climb until they peak, and when the sex difference in prevalence widens [40]. Thus improved, personalized treatment is likely to be most impactful at these ages. Adolescents of these ages are also likely to own a smartphone—mobile sensing research assumes typical phone use, and research found that adults often did not carry study phones with them or use them as they would use personal phones [31]. Exclusion criteria include the following: (1) if receiving pharmacotherapy or psychotherapy for depression, the type or dose of therapy or dosage of medication has not been stable for at least the past 4 weeks; (2) current psychotropic medication taken as needed that could influence mood and related symptoms; (3) acute suicidality (ie, past-month method, plan, intent, access, perceived ability and probability of carrying out attempt); (4) past-year suicide attempt or serious nonsuicidal self-injury (NSSI), or history of suicide attempt over a year ago and not currently receiving mental health services; (5) history of mania or psychosis; (6) adolescent is not fluent in English; and (7) parent or legal guardian is fluent in neither English nor Spanish. Before December 2022, adolescents who reported any history of suicide attempt were excluded, and before February 2022, adolescents who reported any history of serious NSSI were also excluded, and the cutoff score was ≥20 on the CES-D to be included in the study. However, we broadened the inclusion criteria to address challenges with recruitment.

To account for expected attrition of 15%, 59 participants will be recruited from multiple sources, which include a university youth mental health clinic and local middle and high schools within south Florida in the United States, as well as social media posts, advertisements, and community referrals. Interested families are contacted to complete a brief phone screen where they will be assessed for eligibility. Adolescents are screened with the CES-D, which has been validated extensively with adolescents aged between 12 and 18 years [41], including those of Hispanic ethnicity [42], expected to comprise roughly 70% of our sample. The CES-D can discriminate severity at lower levels of depression [43], and 40%-50% of adolescents are likely to meet our cutoff score of ≥16 [44], making this an appropriate measure to select a sample of adolescents with elevated symptoms of depression.

### Ethical Considerations and Informed Consent

Study procedures were approved by the Florida International University Institutional Review Board in April 2020 (IRB-20-0257) and renewed annually thereafter. Due to the sensitive nature and large amount of data collected by the smartphone app from a vulnerable youth population, informed consent is required from both legal guardians (if applicable) of adolescents under the age of 18 years to participate in the study. Adolescents aged 18 years (ie, legal adults in the United States), as well as caregivers, provide informed consent for themselves to participate.

### Safety Planning and Daily Safety Surveys

All families who consent to participate in the study are given brief psychoeducation about depression. Given the increased risk of adolescents with depression having thoughts of suicide [45], adolescents also complete a suicide risk assessment and safety plan [46] with a member of the research team during baseline assessment. The adolescent’s assessment and safety plan are then reviewed with the participating caregiver, who is engaged to provide additional information and feedback and encouraged to support the adolescent in following the safety plan whenever needed, and a copy of the safety plan is provided to the family. Adolescents with elevated risk are excluded from the study and connected to crisis support services as appropriate. In addition, all adolescent participants complete a daily safety survey as part of our EMA procedures. Adolescents are asked whether they have experienced abuse or suicidal ideation since the last survey. Endorsement of abuse will prompt an automated reminder to call 911 or contact a trusted adult, as well as a call from the research team to the youth within 24 hours. Endorsement of suicidal ideation will prompt an automated reminder to use the safety plan or call 911, as well as a call from the research team to the caregiver within 24 hours. Families are instructed not to use the safety survey or other mobile data collected as a way to seek help in an emergency, as it is not feasible for data to be reviewed frequently throughout the day. The adolescent and parent participating in the study then complete a brief quiz to demonstrate that they understand risk procedures in the event that the adolescent reports suicidal ideation or abuse on their daily surveys. All research team members who interact with families receive training and ongoing supervision by the principal investigator, a licensed clinical psychologist, in risk and mandated reporting procedures. In addition, research team members are instructed to contact the principal investigator or an on-call licensed mental health clinician at the university mental health clinic shortly after receiving any report of suicidality or possible abuse from adolescents to ensure that appropriate steps are taken to ensure the safety of the adolescents.

### Data Collection

All assessments take place remotely [47]. The wrist-worn actigraph is mailed to participants in advance of the baseline assessment. During the baseline assessment, caregivers and adolescents who meet all inclusion criteria complete a series of questionnaires measuring demographics, depressive symptoms, and depressogenic processes (Table 1). Adolescents are guided to install the Effortless Assessment of Risk States (EARS) mobile app [27,48] on their smartphones, and, for the next 28 days, the app collects EMA and mobile sensor data, and adolescents wear the actigraph device. EARS was developed for both iPhones and Android phones, is updated to maintain compatibility with newer operating systems, and uses rigorous data security features*.* During the 28-day smartphone and actigraph period, the app presents feedback to adolescents about their survey completion and bonuses earned and uses gamification features (eg, graphics and trophies) to reinforce adherence. Smartphone data will be continuously uploaded to a cloud server and monitored; those who uninstall the app or miss consecutive surveys will be called or texted and offered assistance. Families receive up to US $191 for study completion, including bonuses for completion of all surveys per day and 80%-90% overall adherence to EMA and actigraph procedures. At follow-up, families complete measures and a user feedback interview (Table 1). EARS is then uninstalled, and the actigraph is mailed back to the research team.

**Table 1:** Measures completed by adolescents and caregivers at baseline and follow-up assessment.

Measures						Baseline		Follow-up
**Demographics, ethnic identity, and pubertal development**								
			Demographic information and adolescent self-reported smartphone habits			Adolescent and caregiver		—^a^
			Multigroup Ethnic Identity Measure [49,50] validated with diverse samples of adolescents and adults [51]			Adolescent and caregiver		—
			Pubertal Developmental Scale [39,52]			Adolescent		—
**Adolescent depression and related symptoms, and caregiver depression**								
				Center for Epidemiologic Studies Depression Scale [39] measures adolescent and caregiver self-reported depression	Adolescent and caregiver		Adolescent and caregiver	
				Youth Self-Report and Child Behavior Checklist [53] measures internalizing and externalizing problems	Adolescent and caregiver		Adolescent and caregiver	
				Affective Reactivity Index [54] measures irritability	Adolescent and caregiver		Adolescent and caregiver	
				Children’s Reports of Sleep Patterns [55] measures sleep problems, validated with polysomnography	Adolescent		Adolescent	
				Top problems assessment [56] measures problems youth or caregivers identify as most important	Adolescent and caregiver		—	
**Use of services and impact of COVID-19**								
		Adolescent Medication, Therapy, and Services assesses mental health service use			Adolescent and caregiver		Adolescent and caregiver	
		Epidemic-Pandemic Impacts Inventory [57] measures the impact of the COVID-19 pandemic on various domains of personal and family life			—		Caregiver	
		University of Florida Life Space Mobility rates the frequency of movement before and after the COVID-19 pandemic			—		Adolescent	
		Environmental Influences on Child Health Outcomes COVID-19 Scale measures the impact of the COVID-19 pandemic on school, stress, activity levels, and other domains			—		Adolescent	
**Adolescent depressogenic processes and Research Domain Criteria constructs**								
	Rumination Response Scale [58] measures negative perseverative cognitions					Adolescent		Adolescent
	Automatic Thoughts Questionnaire [59] measures negative automatic cognitions					Adolescent		Adolescent
	Behavioral Activation/Inhibition Scale [60] measures reward responsiveness and potential threat response					Adolescent		Adolescent
	Responses to Stress Questionnaire [61] measures coping and involuntary stress responses. Adolescents select the version of the questionnaire that corresponds to the stressor that is most applicable to them. Adolescents can select from peer stress, maternal depression, paternal depression, family stress, academic problems, COVID-19, or financial problems					—		Adolescent
	Multidimensional Scale of Perceived Social Support [62,63] measures affiliation with friends, family, and significant other					Adolescent		Adolescent
	Berkeley Expressivity Questionnaire [64] measures social communication through the production of facial expression					Adolescent		Adolescent
	Snaith-Hamilton Pleasure Scale [65] measures anhedonia					Adolescent		Adolescent
Feedback interview about ecological momentary assessment, mobile sensing, and actigraphy. Rate how enjoyable, easy to use, disruptive, acceptable; privacy concerns and changes in phone use during the study; describe likes or dislikes, suggested improvements, and health-related apps currently used						—		Adolescent and caregiver

^a^Measures not given to adolescents and caregivers.

### EMA

Symptom surveys are administered 5 times a day on weekdays and 6 times a day on weekends, over 28 consecutive days. Prompts are delivered randomly within 2-hour blocks in the morning (9 AM-11 AM), around noon (11 AM-1 PM), early afternoon (1 PM-3 PM), late afternoon (3 PM-5 PM), and evening (5 PM-7 PM), with a late evening (7 PM-9 PM) block on weekends. Each block includes a 0.5-hour buffer to separate adjacent surveys. A similar sampling schedule has been used successfully (>80% compliance) with adolescents [24]. Adolescents rate on a 0-100 visual analogue scale 21 items adapted from previous studies [11,12,25]: depressive symptoms ("felt down or depressed," "felt hopeless," "felt irritable or cranky," "felt like you don’t enjoy things," "felt bored/uninterested in things," "felt worthless or guilty," "had difficulty concentrating," "felt tired," "felt restless"), depressogenic processes ("dwelled on the past," "avoided people," "avoided activities," "procrastinated"), negative affect/anxiety symptoms ("felt angry," "felt worried," "felt nervous or anxious," "felt afraid," "felt tense in your muscles"), and positive affect ("felt happy or positive," "felt energetic," "felt enthusiastic"). Once per day, adolescents also report their attempted sleep time, sleep onset latency, and wake time; their subjective sleep quality on a 0-100 visual analog scale; and whether they had a “fixed” or “free” wake-up (by alarm or caregivers, noise, or naturally).

### Mobile Sensing

We selected 12 sensor variables to be analyzed based on previous literature [30,32,35,37]. These include raw data on battery status, calls (frequency×duration), as well as extracted features on mobility (percentage of time spent at home and distance traveled from GPS), physical activity intensity from accelerator, sleep duration and efficiency (from ambient light and accelerometer), and natural language use in social communication (from all text typed into email, text, and social media apps, captured by a keyboard logger). Specifically, we will assess word count and word sentiment through the Linguistic Inquiry and Word Count [66] Positive Emotion and Negative Emotion dictionaries, and the use of first-person pronouns (eg, “I”) and absolutist words (eg, “never”) associated with mental health [67-69], in English and Spanish.

### Actigraphy

Adolescents wear on their nondominant wrist a research-grade actigraph, a wearable wrist monitor used to capture and record continuous, high resolution physical activity and sleep and wake information. The wGT3X-BT device (ActiGraph) is widely used in research studies and has been validated against indirect calorimetry and polysomnography [22,23]. It also features a light sensor; thus, this model was initially used with participants. However, feedback interviews from early in the study revealed a strong preference for a less conspicuous device to avoid drawing unwanted attention. A total of 15 adolescents wore the wGT3X-BT model, with an average wear time of only 74.3% for the 13 adolescents who completed 28 days of actigraphy. To maximize adherence to wearing the actigraph, we replaced the initial model (encased in a bright red plastic shell) with the newer GT9X Link model (ActiGraph), which resembles a black smartwatch with a digital display of the time, in August 2022. Since then, 24 adolescents have used or are using the GT9X Link model, resulting in an increased average wear time of 80.2% for the 21 adolescents who completed 28 days of actigraphy using this model. Both models use a triaxial accelerometer and ActiGraph’s proprietary filtering algorithm to assess total sleep time, sleep latency onset, wake after sleep onset, sleep efficiency, sedentary bouts, and physical activity intensity in participants’ natural environments, and were designed to give comparable results. The primary differences between the wGT3X-BT model and the GT9X Link model are that the GTX9 Link model has a liquid-crystal display and a gyroscope, and the other model does not, whereas the wGT3X-BT has an ambient light sensor, and the other model does not. The data will be analyzed with the ActiLife companion software using the Sadeh algorithm to distinguish between sleep and wake states [70].

### Data Analysis

#### Sample Size

The numbers of observations (*t*=148) and participants (n=50) were estimated based on previous research using similar idiographic models [4,6]. Given the mean response rate (78%) in smartphone EMA with youth [71], 115/148 observations per adolescent are expected; thus, up to 23 EMA items can be included in an idiographic model with ≥5 observations per item.

#### Aim 1 Analyses: Idiographic Dynamic Models of EMA Data to Identify Person-Specific Drivers

EMA will yield a multivariate time series for each adolescent to be submitted to network analysis [11,12]. The graphical vector autoregressive (VAR) model will be used to identify contemporaneous and lag-1 directed effects. The graphical VAR function in R [72] models the autocorrelations and cross-predictions in a lag-1 VAR model as directed paths in the temporal portion of the network model, and the residual correlations as associations in the contemporaneous network. Regularization is applied through the least absolute shrinkage and selection operator (LASSO) algorithm, and model selection is performed by the extended Bayesian information criterion [73]. Hypotheses are supported if ≥50% adolescents (1) have contemporaneous networks in which ≥1 item has greater strength centrality at a given time *t*, (2) temporal networks in which ≥1 item has greater outstrength (ie, time *t* values that strongly predict other items at time *t*+1), and (3) if items with the greatest centrality and outstrength vary across adolescents. Because idiographic networks are a new area, there is no established threshold for identifying drivers and distinguishing significant differences among adjacent-ranked items for the same adolescent, other than selecting items with a CI that does not include zero. We will thus explore and consider using novel methods that may facilitate the identification of drivers. For example, a recently developed approach that ranks the items by their controllability (ie, how efficient it is to intervene on each item, taking into account downstream changes in the symptom network [74]) might be helpful for addressing this aim if it can be extended from group analyses to idiographic analyses. In addition, results may not generalize beyond the items in the measure used to generate the network, prompting recommendations to avoid multiple items measuring the same construct and to examine latent variables before estimating networks [75]. Person-specific dynamic factor analysis is another approach to identifying person-specific drivers by examining latent variables of symptoms that predict other latent variables of symptoms over time [76].

#### Aim 2 Analyses: Validation of Mobile Sensing to Identify Person-Specific Drivers

First, we will sample mobile sensor and actigraph data collected during the 15-minute intervals just before and just after each EMA response. (2a) Because of our idiographic focus and previous research showing high between-subject heterogeneity [34], N-of-1 correlations will be computed with the equivalent or conceptually similar actigraph and EMA measures: (1) sleep duration and efficiency, physical activity intensity with EMA items “felt energetic,” “felt tired” (2) communication word count and calls with EMA item “avoided people.” Our a priori thresholds for correlations are *r*=0.32 (minimum acceptable), *r*=0.50 (moderate), and *r*=0.71 (strong), corresponding to 10%, 25%, and 50% of overlapping (shared) variance. Among the various metrics, we believe that overlapping variance represents the degree to which the EMA, sensor, and actigraph data signals stem from the same data-generating process. Moderate to strong correlations support the utility of sensor-measured behaviors as proxies for self-reported tiredness, energy, and social interaction, providing empirical support for replacing these EMA items in idiographic symptom network models. (2b) Second*,* we will assess the 12 mobile sensor variables as predictors of self-reported mood (“felt down or depressed,” “felt hopeless,” “felt irritable or cranky”) and anhedonia (“felt bored/uninterested in things,” “felt like you don’t enjoy things”) using random forests [77], which has been used successfully to analyze mobile sensor data for N-of-1 models [32]. The 5 EMA items will be assessed as separate outcomes in N-of-1 models, a high *R*^2^ indicates that the model closely predicted and could substitute the EMA scores. The same *R*^2^ thresholds of 10%, 25%, and 50% overlapping variance will be used. The robustness of these predictions is assessed with repeated sampling of 100 random training-test data splits, with a 70/30 split of training and test data per sample [32]. This approach accommodates nonlinear relationships and complex interactions, minimizes overfitting the data, and selects the most important predictors [78]. Moreover, random forests perform well in smaller samples without hyperparameter tuning [79] and when the number of trees does not exceed the number of observations [80,81]. Random forest is an ensemble method combining bootstrapped data sets into a final model, and although predictor importance is indicated, the lack of regression coefficients can be challenging for interpretation [32]. Thus, if necessary, we propose an alternative with similar advantages (eg, selects important variables, minimizes overfitting)—elastic net regularization. A k-fold cross-validation with 20 folds will be used to select the optimal model with the minimum mean cross-validated error. This approach produces fewer prediction errors than comparable methods (eg, LASSO and ridge regression).

#### Aim 3 Analyses: Exploration of Baseline Predictors of Person-Specific Drivers

From the idiographic models in aim 1, ≥50% of adolescents are expected to have drivers (1 or 2 out of 18 EMA symptoms with the greatest strength centrality and outstrength). A wide range of baseline adolescent characteristics (demographic, developmental, and clinical) will be examined as predictors of driver status (yes vs no) in a between-subjects (all participants) random forest classification model for every symptom. An area under the curve >0.50 indicates that the predictors can predict driver status of a given symptom better than chance. The robustness of classification is assessed with a 70/30 split of participants into a training set and a testing set [32]. If necessary, we propose elastic net regularization as an alternative due to the reasons described in aim 2, and good performance even though the number of participants is small compared to the number of predictors [82].

## Results

Recruitment began in May 2021. As of October 2023, we have contacted 1069 families; 22% (n=235) expressed interest in participation and completed a phone screen, of whom 36% (n=84) were eligible to enroll in the study. Of the 84 eligible families, 70% (n=59) provided informed consent, 63% (n=53) completed a baseline assessment, and 46% (n=39) met all inclusion criteria. Of the 25 families who were eligible but elected not to participate in the study, 52% (n=13) did not provide a reason, 28% (n=7) were unable to be reached, 16% (n=4) were not comfortable with the amount of data collected or frequency of surveys, and 4% (n=1) were only able to get consent from one of their 2 caregivers in order to participate. A total of 6 families provided informed consent but did not continue with the study, of whom 66% (n=4) did not provide a reason, 17% (n=1) stated their schedule was not compatible with the study, and 17% (n=1) were unable to be reached. During baseline assessment, 17% (n=14) of families were excluded from the study, of whom 36% (n=5) were excluded for acute suicidality, and the remaining 64% (n=9) were excluded due to a history of serious NSSI, suicide attempt, or both. Of the 39 included families, 85% (n=33) completed the 28-day smartphone data collection period and follow-up study visit, 8% (n=3) lost the actigraph, 5% (n=2) stopped participation early, and 3% (n=1) is currently in the process of completing the study.

## Discussion

### Overview

In this protocol report, we describe the methods of a study using smartphone-based EMA and passive sensing with the goal of identifying person-specific drivers of depression in adolescents. We have provided a status update on data collection for this ongoing study. Of the 59 adolescents targeted for recruitment, two-thirds (n=39) have started the study, and half have completed all phases of the study (n=33). After data collection is complete, we plan to address the 3 specific aims of this study. First, we will investigate whether there is evidence for person-specific drivers of depression among this sample of adolescents. That is, we will identify symptoms that strongly predict change in other symptoms for each adolescent and examine whether these driver symptoms differ across adolescents. Second, we will validate mobile sensor data against EMA and actigraph measures. We will assess whether smartphone-measured sleep, physical activity, and social interaction variables are correlated with conceptually similar actigraph-measured variables and EMA responses, and whether mobile sensor data can predict EMA responses about the key depressive symptoms of low mood and anhedonia within each adolescent. Third, we will explore whether baseline adolescent characteristics can predict which symptoms are drivers for which adolescents.

Empirical findings suggesting that person-specific drivers of adolescent depression exist would offer a plausible explanation for individual differences in clinical presentation and treatment response in this population. These findings would also support the need for a personalized approach to assessment and intervention, where treatments or their component modules are selected and sequenced to target each adolescent’s specific drivers [14,15]. Indeed, emerging research suggests that depressed youth display heterogeneous responses to individual CBT modules [83,84]. Moreover, the particular symptoms identified as drivers in our adolescent sample could be compared to those identified in adult samples. Differences in driver symptoms between adolescents and adults may provide clues as to why depressed adolescents do not benefit as much from psychotherapy as do adults [6], thereby informing the generation of hypotheses about developmental considerations in the treatment of adolescent depression for future testing.

Our efforts to validate mobile sensor data for predicting depressive symptoms in adolescents will add to the small but growing literature on this topic [33-36]. Recent studies have demonstrated promising results in predicting depressive symptom scores [33,35] and diagnosis [36] using smartphone and wearable sensor data. One study [34] found good prediction of elevated anger and anxiety but more modest prediction of sadness from mobile sensor data—a finding that is particularly relevant to this study’s focus on assessing and predicting the severity of individual depressive symptoms. Accurately predicting individual symptom severity with mobile sensor data obtained with minimal user effort can make the assessment of person-specific drivers more feasible to use with adolescents in clinical practice.

If adolescent characteristics measured at baseline are found to predict each adolescent’s driver symptoms reasonably well, then a preliminary assessment of drivers could be made early in the evaluation, shortening the lag time from intake to the initiation of treatment. The month-long smartphone tracking phase may be used to confirm the preliminary assessment results or perhaps even be skipped entirely. Although identifying drivers focuses on intrapersonal symptom dynamics, examining between-subject characteristics that predict those drivers could shed light on interpersonal differences in psychopathology and begin to illuminate subgroups of adolescents with different optimal treatment targets. A future direction is to use the data generated by this study to inform the development and testing of a scalable, low-burden smartphone-based tool and treatment algorithm to select and sequence therapy modules targeting person-specific drivers in depressed adolescents with potential public health impact.

### Limitations

This study is recruiting participants from the community who generally have mild depressive symptoms and low risk of suicide and self-harm. We focused on this population due to practical reasons relating to recruitment and our capacity for responding to emergent crises. However, adolescents with severe depressive symptoms and higher risk of suicide and self-harm may be in greatest need of effective treatment. In order to inform personalized treatment for the adolescents who may benefit the most from it, further research on person-specific drivers of depression needs to be conducted with this population.

In addition, this study cannot establish whether identified drivers cause changes in other depressive symptoms, only whether they are associated with changes in depressive symptoms. It is thus possible that a third variable is causing changes in both driver and nondriver depressive symptoms. Moreover, contemporaneous networks cannot determine the direction of association between central symptoms and other symptoms; changes in other symptoms may in fact precede changes in central symptoms, or the association may be bidirectional. A related concern is that the temporal networks’ ability to capture directional change is constrained by the EMA sampling intervals of 2 hours—directional change that occurs during a shorter or much longer time frame will not be detected.

Furthermore, data were collected during the COVID-19 pandemic, a time when adolescents had to deal with multiple stressors, including fears about themselves or their loved ones being infected, losses (eg, of life, job, or relationships) experienced by themselves or family members, disruptions to schooling and social activities, and isolation at home due to safe distancing measures [85]. Studies have documented increased depressive symptoms and loneliness, as well as poorer academic functioning among adolescents during the pandemic, with notable heterogeneity in pandemic impact attributed to differential access to resources and preexisting vulnerabilities [86]. To assess how the pandemic may influence the associations between adolescents’ behavior assessed by mobile sensors and their depressive symptoms, we have included several measures of pandemic impact on adolescents and their parents.

### Conclusions

As one of very few studies examining depressive symptom networks among adolescents, this study is expected to offer novel insights into the structure and dynamics of depressive symptomatology during a sensitive period of development. A better understanding of what drives depression during this high-risk, high-impact window of development could lead to assessments and interventions that better address developmental and individual differences, ultimately producing greater benefit over the life course. Finally, this study is expected to contribute to the growing literature supporting a shift from standardized to personalized assessment and treatment of adolescent depression.

## Abbreviations

CBT: cognitive behavioral therapy
CES-D: Center for Epidemiologic Studies Depression Scale
EARS: Effortless Assessment of Risk States
EMA: ecological momentary assessment
LASSO: least absolute shrinkage and selection operator
NSSI: nonsuicidal self-injury
VAR: vector autoregressive

## Data Availability

The data sets generated during this study are available from the National Institute of Mental Health Data Archive at the National Institutes of Health. The National Institute of Mental Health Data Archive is a collaborative informatics system created by the National Institutes of Health to provide a national resource to support and accelerate research in mental health. Data set identifier is 10.15154/88w5-4029. This manuscript reflects the views of the authors and may not reflect the opinions or views of the National Institutes of Health.
